# Spatial learning and memory deficits induced by prenatal glucocorticoid exposure depend on hippocampal CRHR1 and CXCL5 signaling in rats

**DOI:** 10.1186/s12974-021-02129-8

**Published:** 2021-04-02

**Authors:** You Zheng, Yan-Min Zhang, Zheng-Shan Tang, Jian-Kui Du, De-Wei Guo, Yong-Jun Xu, Hui Sheng, Jian-Qiang Lu, Xin Ni

**Affiliations:** 1 Department of Gynecology and Obstetrics and Research Center for Molecular Metabolomics, Xiangya Hospital Central South University, Changsha, 410008 China; 2 Department of Physiology, Navy Medical University, Shanghai, 200433 China; 3 Laboratory of Cell Engineering, Institute of Biotechnology, Beijing, 100071 China; 4 School of Kinesiology, Shanghai University of Sport, Shanghai, 200438 China; 5 National Clinical Research Center for Geriatric Disorders, Xiangya Hospital Central South University, Changsha, 410008 China

**Keywords:** Glucocorticoids, Programming, Corticotropin-releasing hormone, Spatial learning and memory, CXCL5, Pregnancy

## Abstract

**Background:**

Prenatal synthetic glucocorticoid (sGC) exposure increases the susceptibility to cognitive and affective disorders in postnatal life. We previously demonstrated that prenatal sGC exposure results in an increase in corticotropin-releasing hormone (CRH) receptor type 1 (CRHR1) expression in the hippocampus of rats, and CRHR1 is involved in synapse formation via regulation of C-X-C chemokine ligand 5 (CXCL5) in hippocampus. We sought to investigate that the roles of CRHR1 and CXCL5 in learning and memory impairment caused by prenatal sGC exposure.

**Methods:**

Pregnant rats were administered with saline or dexamethasone (DEX) from gestational day (GD) 14 to GD21. DEX offspring at 2-day old were treated with saline and CRHR1 antagonists (antalarmin and CP154526) for 7 days. Some DEX offspring received intra-hippocampal injection of AAV9 carrying CXCL5 gene. Spatial learning and memory was assessed by Morris water maze test. Immunofluorescence analysis was applied to show synapsin I and PSD95 signals in hippocampus. Synapsin I and PSD95 protein level and CXCL5 concentration were determined by western blotting and ELISA, respectively. Organotypic hippocampal slice cultures were used to investigate the effect of DEX on CXCL5 production in vitro.

**Results:**

Both male and female DEX offspring displayed impairment of spatial learning and memory in adulthood. Synapsin I and PSD95 signals and CXCL5 levels were decreased in DEX offspring. DEX offspring with antalarmin and CP154526 treatment showed improved spatial learning and memory. Antalarmin and CP154526 treatment increased synapsin I and PSD95 signals and CXCL5 concentration in hippocampus. Bilaterally hippocampal injection of AAV9 carrying CXCL5 gene improved the spatial learning and memory and increased CXCL5 concentration and synapsin I and PSD95 levels in hippocampus. DEX dose-dependently suppressed CXCL5 production in cultured hippocammpal slices, which was prevented by antalarmin treatment.

**Conclusion:**

CRHR1 and CXCL5 signaling in the hippocampus are involved in spatial learning and memory deficits caused by prenatal DEX exposure. CRHR1 activation contributes to decreased CXCL5 production in hippocampus induced by prenatal DEX treatment. Our study provides a molecular basis of prenatal GC exposure programming spatial learning and memory.

## Background

During pregnancy, glucocorticoids (GCs) are critical for fetal organ development and maturation including the lungs and brain [1]. Pregnant women at the risk of preterm delivery usually therefore receive treatment with synthetic GC (sGC) in order to promote the development of fetal organs and impede preterm delivery associated morbid symptoms, such as respiratory distress syndrome and intra-ventricular hemorrhage [2, 3]. Epidemiological studies have indicated that prenatal exposure to GCs by either maternal stress or sGC treatment leads to increased susceptibility to cardiovascular, metabolic, and neuropsychiatric disorders in adulthood [4–7], which has been confirmed by many studies in animals [8–11]. Moreover, impaired learning and memory have been shown in the models of rodents and primates with prenatal exposure to GCs [9, 12, 13].

Hippocampus is the key brain region in cognitive, affective, and behavioral functions and is a highly plastic structure that is sensitive to the effects of steroid hormones [14–16]. A number of studies have demonstrated that prenatal GC exposure alters genome-wide transcription in the hippocampus, thereby leading to long-term effects [17–19]. Our previous study has shown that corticotrophin-releasing hormone (CRH) and CRH receptor type 1 (CRHR1) genes are upregulated in the hippocampus of the rats with prenatal sGC exposure [20]. Emerging evidence indicates that CRH is the key mediator in stress-related affective disorders and impaired learning and memory [21–28]. These effects are at least in part associated with CRH modulation of synaptic formation. For instance, CRH mediates stress-induced rapid loss of apical dendritic spines in CA1 and CA3 pyramidal cells of hippocampus [21, 22]. The absence of CRHR1 prevents the detrimental effects of chronic stress on spatial memory as well as dendritic arborization of CA3 neurons [26, 27]. More recently, using in vitro models, we have found that CRH suppresses synaptic formation of hippocampal neurons via inhibition of C-X-C chemokine ligand 5 (CXCL5) output from glia [29].

Based on the above background, we hypothesized that upregulation of CRH/CRHR1system contributes to the impairment of learning and memory caused by prenatal sGC exposure, and CXCL5 might be involved in these effects. To test it, we set up of series of experiments to firstly confirm that prenatal dexamethasone (DEX) treatment resulted in impairment of spatial learning and memory and a decrease in synaptic network and CXCL5 level in the hippocampus. Then, we explored the effects of blocking CRHR1 signaling on spatial learning and memory and synaptic signals in DEX offspring. Finally, using AAV9 system and organotypic hippocampal slice culture, we elucidated that CXCL5 plays an important role in impaired spatial learning and memory induced by prenatal DEX exposure.

## Material and methods

### Animals

All animal procedures were carried out in accordance with the guidelines for the use of laboratory animals published by the People’s Republic of China Ministry of Health [May 2016], with the approval of the Ethical Committee of Experimental Animals of Navy Medical University and Ethical Committee of Xiangya Hospital Central South University. Procedures were designed to minimize the number of animals used and their suffering. Adult Sprague-Dawley rats, weighing 220±20g, were purchased from Shanghai SLAC Laboratory Animal Co., Ltd. (Shanghai, China). The rats were housed with regular light-dark cycles (lights on at 7:00a.m., lights off at 7:00p.m.) under controlled temperature (22±2 °C) and humidity (50±10%) and were given with standard diet and water ad libitum.

### Experimental design

**Fig. 1:**
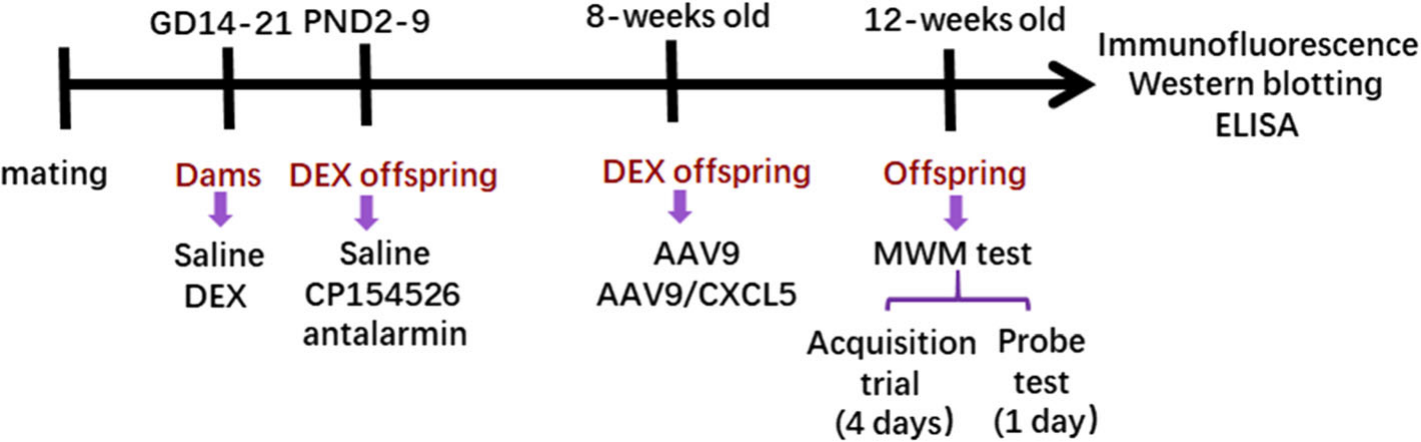
Timeline of experimental procedures. Pregnant rats were treated with (s.c) dexamethasone (DEX) or vehicle (saline) from GD 14–21. Some DEX offspring at 2-days old were injected with (s.c) saline, CP154526 or antalarmin for 7 days. Some 8-week-old DEX offspring were received intra-hippocampal injection of AAV9 or AAV9/CXCL5. MWM test was performed in offspring of 12 weeks old with a 5-day test. Then, the rats were sacrificed and hippocampal tissues were collected for immunofluorescence, western blotting, and ELISA

The animal model of prenatal exposure to sGC was established as described previously [28]. Figure 1 illustrates the timeline of experimental procedures. Briefly, rats were mated with confirmation by microscopic analysis of vaginal smears for the presence of sperm in the next morning. Pregnant rats were assigned randomly to control and DEX group (*n*=12 in each group). Dexamethasone-21-phosphate disodium salt (Sigma-Aldrich, St. Louis, MO) was dissolved in saline to achieve 0.13 mg/kg body weight in 100μl. Rats received 0.13 mg/kg dexamethasone-21-phosphate disodium salt (equal to 0.1 mg/kg DEX, DEX group) or saline (control group) once a day from GD14-21. Vehicle- or DEX-exposed offspring were weaned on postnatal day 21 (PND21) and reared in mixed social groups until 12 weeks old. Twelve males and 12 females were randomly picked up from each group for behavior test at 12 weeks old. To minimize litter specific effects, only 1 male and 1 female were taken from each litter for inclusion in each treatment group. After behavior test, the rats were sacrificed by anesthetization with intraperitoneal injection of 100 mg/kg pentobarbital. The hippocampus was dissected and frozen on dry ice. For the study of the effect of CRHR1 antagonists on spatial learning and memory in prenatal DEX rats, 72 males and 72 females of a 2-day old DEX offspring were randomized to one vehicle group, two CP154526 groups, and two antalarmin groups (*n*=12 in each group) in each sex. They were subcutaneously administered with CP154526 (Tocris Cookson, Inc., Ellisville, MO) at the dose of 10 and 30mg/kg/day, or antalarmin (Sigma-Aldrich, St. Louis, MO) at 10 and 30 mg/kg/day for 7 days. The rats of vehicle group were injected with saline. The dosages of CP154526 and antalarmin as well as the intervention time were chosen according to literatures [30, 31] and our previous study [20]. At 12 weeks old, these rats underwent behavioral tests and then were sacrificed by anesthetization and collection of brain tissues. Some of them were deeply anesthetized by intraperitoneal 100mg/kg pentobarbital injections and perfused with saline and fixed with paraformaldehyde. The hippocampal tissues were dissected for immunofluorescence analysis. In addition, some DEX offspring at 8 weeks old were used for intrahippocampal injection of AAV9 vector or AA9 carrying CXCL5 gene. Four weeks after injection, animals were subjected to tests of spatial learning and memory. After behavioral test, the rats were sacrificed for tissue collection.

### Morris water maze (MWM) test

Spatial learning and memory of rats was assessed by MWM test [32]. Briefly, the test was performed in a 1.6-m diameter pool with a 12-cm diameter platform placed in the southeastern quadrant of the pool. The pool was filled with water (22±2 °C) up to 40-cm-depth. A standard 5-day Morris water maze test, which consists of 4 days of hidden platform test (the acquisition trial) plus a probe test (24 h after the last hidden platform test), was performed by a researcher blinded to treatment groups. The 4 days of hidden platform test included spatial acquisition training and testing sessions, which included four sessions per day at an inter-session interval of 60 min. In this test, the escape platform was hidden 2 cm below the water surface. The first day of acquisition trial was named as day 1. Rats were trained to escape from drowning by climbing onto the hidden platform, and they were allowed to swim for maximum trial duration of 60s. If the animal failed to reach the platform within 60 s, the escape latency of “80 s” was awarded. Rats were allowed to stay on the platform for 20 s before being removed. Swimming speed and escape latency were analyzed using a motion detection software. The escape latency from all four sessions was averaged to calculate the escape latency for each day. Probe test was carried out 24 h after the last trial of day 4. In the probe test, the platform was removed from the previous quadrant and each rat was placed into the pool from the opposite quadrant. The probe trial consisted of a 30-s free swim period without a platform. The time swum or stayed in the target quadrant of the rats was recorded.

### Immunofluorescence analysis

The brain tissues were fixed in 4% paraformaldehyde in phosphate-buffered saline (PBS) for 24h at 4°C and then washed twice in PBS. The samples were then dehydrated through an ethanol series, cleared by soaking in xylene, embedded in paraffin, and sectioned into 4μm. Slides containing paraffin sections were deparaffinized in xylene and rehydrated through an ethanol series, then incubated in Citrate Antigen Retrieval Solution (Beyotime, P0081) at 90 °C for 20min. The sections were washed with PBS and incubated with 10% BSA for 2h and then were incubated with primary antibodies against synapsin I (AB1543P, Millipore), PSD95 (ab2723, Abcam), CXCL5 (PA5-103851, Invitrogen), GFAP (Ab68428, Abcam), and MAP2 (ab254264,Abcam) at 1:200 dilution in PBS containing 2% BSA overnight at 4°C. Subsequently, the specimens were incubated with anti-rabbit IgG conjugated to Alexa Fluor 488 (A-21206, Thermo Fisher) and anti-mouse IgG Alexa Fluor 594 (R37115, Thermo Fisher) at 1:400 dilution. Staining images were visualized using Leica confocal microscope (Lecia Microsystems Inc., Buffalo, USA). Images of hippocampal slice were transferred to ImageJ software and quantified by measuring the average pixel intensity of the hippocampus.

### Western blot analysis

Hippocampal tissues were homogenized in the presence of lysis buffer consisting of 60 mM Tris-HCl, 2% sodium dodecyl sulfate (SDS), 10% sucrose, 2 mM phenylmethylsulfonyl fluoride (Merck, Darmstadt, Germany), 1 mM sodium orthovanadate (Sigma-Aldrich), and 10 μg/ml aprotinin (Bayer, Leverkusen, Germany). The lysates were quickly centrifuged at 4°C. The supernatant was collected, and protein concentration was assayed using a modified Bradford assay. The samples were then diluted in sample buffer (250 mM Tris-HCl, 4% SDS, 10% glycerol, 2% β-mercaptoethanol, and 0.002% bromophenol blue) and boiled for 10 min. Aliquots of proteins were separated by SDS-PAGE (10%) and subsequently transferred to nitrocellulose membranes by electroblotting. The membrane was blocked in 5% skim milk powder in 0.1% Tris-buffered saline/Tween 20 (TBST) at room temperature for 2h and then incubated with antibodies against synapsin I (AB1543P, Millipore) and PSD95 (ab2723, Abcam) at a dilution 1:1000 overnight at 4°C. After three washes with TBST, the membrane was incubated with the secondary antibodies of horseradish peroxidase-conjugated antibody for 1 h at room temperature. Immunoreactive proteins were detected using the enhanced chemiluminescence Western blot detection system (Santa Cruz) and visualized using Sygene Bio Image system (Synoptics Ltd, UK). To control sampling errors, the ratio of band intensities to β-actin (A5441, Sigma-Aldrich) was obtained to quantify the relative protein expression level.

### Organotypic hippocampal slice culture

Hippocampal slice cultures were performed as previously described [29]. In each independent culture, 6 male newborn rats (P2-3) from same litter of a dam were used. Briefly, after decapitation, rat brains were removed and placed in ice-cold oxygenated low sodium-containing artificial cerebral spinal fluid (containing 248 mM sucrose, 4 mM KCl, 1.25 mM NaH_2_PO_4_, 26.2 mM NaHCO_3_, 1 mM CaCl_2_, 5 mM MgCl_2_, and 10 mM glucose) and then carefully placed on the platform of a tissue chopper and sliced perpendicular to its longitudinal axis using a vibrating microtome (NVSLM1, World Precision Instruments Inc.), with 400 μm thickness of each slice. Slices were transferred to Millicell CM membrane inserts (Millipore, Bedford, MA, USA) in 6-well culture plates. Each well contained 1.2 ml of pre-warmed DMEM (Invitrogen Corp., Carlsbad, CA) containing 20% horse serum (Invitrogen), 10.5 mM glucose, 12.5 mM HEPES, and 55 mM NaHCO_3_ (pH 7.3–7.4). The slices were incubated in a humidified, 5% CO_2_ atmosphere at 37^o^C overnight, and followed by treatments with increasing concentration of dexamethasone-21-phosphate disodium salt in the presence or absence of CRHR1 antagonist antalarmin for 24h. The slices were then harvested for measurement of CXCL5 content.

### Enzyme-linked immunosorbent assay (ELSIA)

The concentration of CXCL5 in hippocampal slices and tissues was determined by commercial Rat CXCL5 ELISA kit (RayBiotech, Inc.) following the manufacturer’s instructions.

### Stereotaxic microinjection of AAV9

AAV9 vector (containing EGFP gene) carrying CXCL5 gene was constructed by Shanghai Sunbio Medical Biotechnology Co., Ltd. The virus titers was 1×10^9^ transduction units (TU)/ml. Intra-hippocampus infusion of AAV9 encoding CXCL5 gene was performed using the method of stereotaxic injections as described previously [20, 33]. DEX offspring at 8 weeks old were anesthetized with 10% chloral hydrate (0.33mg/kg, i.p.) and placed in a stereotaxic apparatus (Stoelting Co., USA). AAV9 vector carrying CXCL5 gene and AAV9 vector were injected bilaterally into the CA1 region of hippocampus (2μl per site) using microliter syringes (Hamilton CO., Reno, NV, USA). The target coordinates of CA1 region were shown as followings: −4.2 mm posterior to bregma, ± 3.0 mm lateral to bregma, and 2.9 mm ventral to the dura. The injection rate was 0.2μl/min by using a syringe pump. As AAV9 carry EGFP gene, the brain tissues were fixed with paraformaldehyde and then series of sections were made to show the injection site and the AAV9 infection efficiency.

### Statistics

Data were presented as mean ± SEM. Statistical Package for Social Sciences (SPSS) software version 16.0 was used. Normal distribution was assessed by Shapiro-Wilk test. Statistical significance was determined according to sample distribution and homogeneity of variance. Statistical comparisons between two groups were determined by two-tailed Student’s *t* test. Data from multiple groups were compared using one-way ANOVA, followed by Bonferroni’s post hoc test. For the data of acquisition, trials were analyzed by the two-way repeated measures ANOVA and adjusted by the Bonferroni test for main effects. Differences were considered significant when *P* < 0.05.

## Results

### Prenatal DEX exposure causes spatial learning and memory impairment

**Fig. 2:**
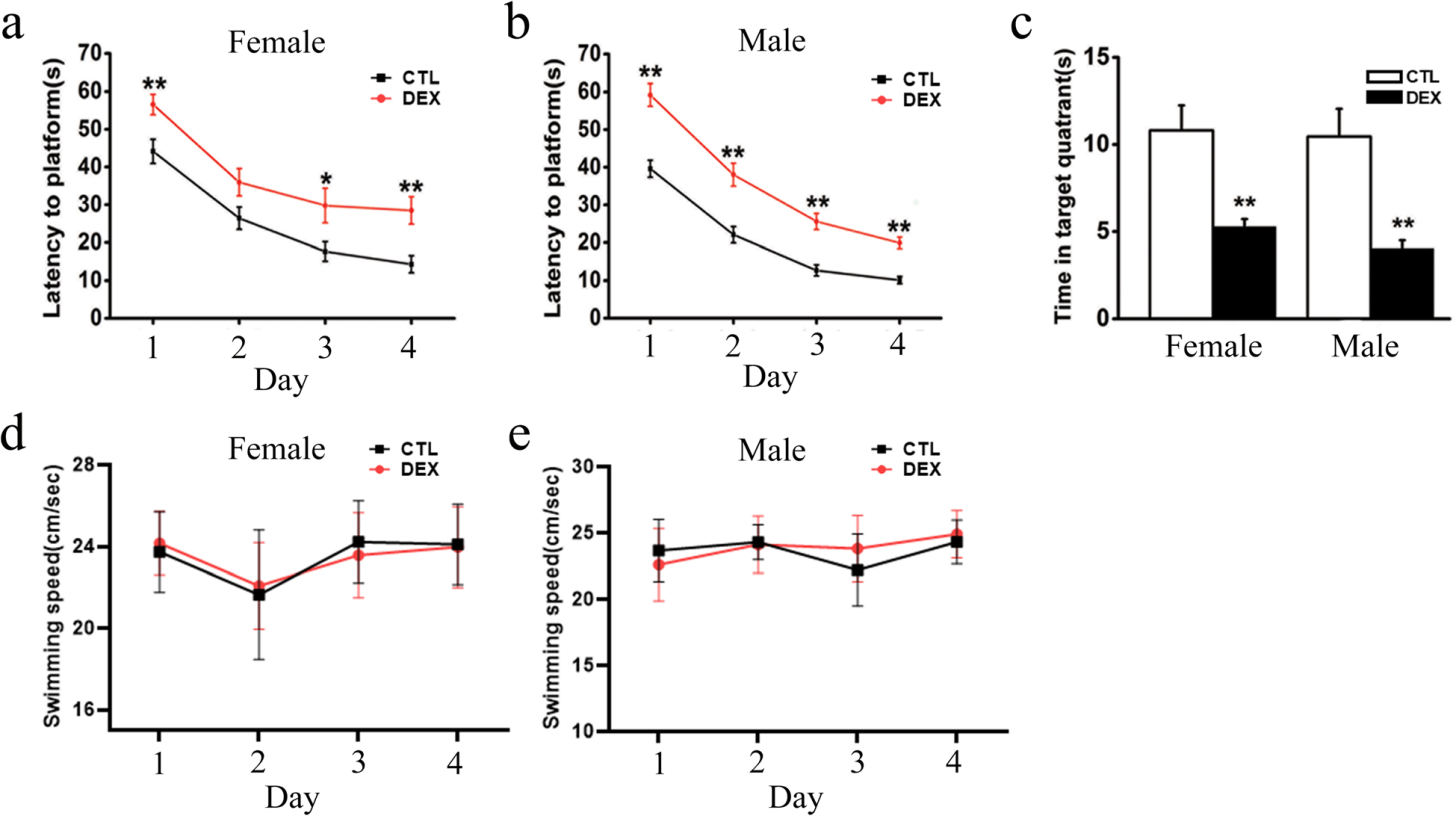
The effects of prenatal DEX exposure on spatial learning and memory. Pregnant rats received injection of dexamethasone or vehicle on GD 14–21. Behavioral tests were performed on 12-week-old offspring. **a**, **b** The escape latencies by that prenatal DEX or control females (**a**) and males (**b**) find hidden platform in the 4-day acquisition trial (**c**), the time by that the rats spent in the platform quadrant in the probe trial. **d**, **e** The swimming speed of DEX offspring and control offspring. Data are presented as mean ± SEM (*n*=12 in each group). *P<0.05, **P<0.01 vs control. CTL: control

As shown Fig. 2a&b, escape latency progressively decreased over 4 days of training in all groups. Both female and male DEX offspring had longer escape latencies than the corresponding control offspring on days 1–4. Compared to the control offspring, the DEX offspring spent a shorter amount of time in the target quadrant on the test day (Fig. 2c). There was no difference in swimming speed among control and DEX offspring groups (Fig. 2d&e), suggesting no motor dysfunction in any rats. These results suggested that DEX offspring have spatial learning and memory deficits.

### Administration of CRHR1 antagonists improve spatial learning and memory in DEX offspring

**Fig. 3:**
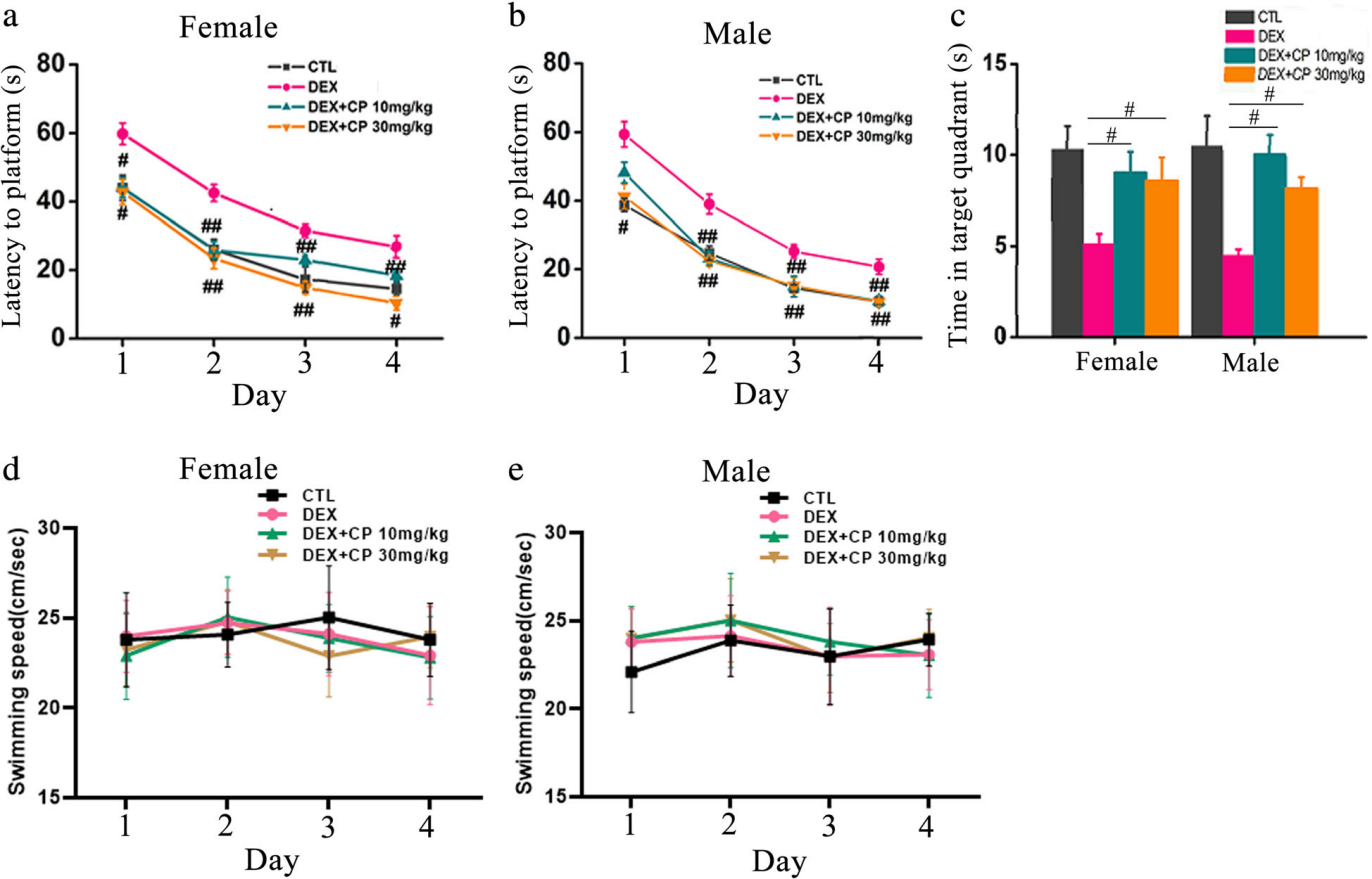
CRHR1 antagonist CP154526 ameliorates spatial learning and memory deficits in DEX offspring. DEX offspring of 2 days old were subcutaneously administered with CP154526 (10mg/kg/day, 30mg/kg/day) for 7 days. Morris water maze test was performed on these rats in adulthood. **a**, **b** The escape latencies of CP154526 treated female (**a**) and male (**b**) DEX offspring in the 4-day platform acquisition training. **c**, the time that spent in the correct platform quadrant in the probe trial after training. **d**, the swimming speed of the rats. Data are presented as mean ± SEM (*n*=12 in each group). #*P*<0.05, ##*P*<0.01 vs DEX. CTL: control, CP: CP154526

**Fig. 4:**
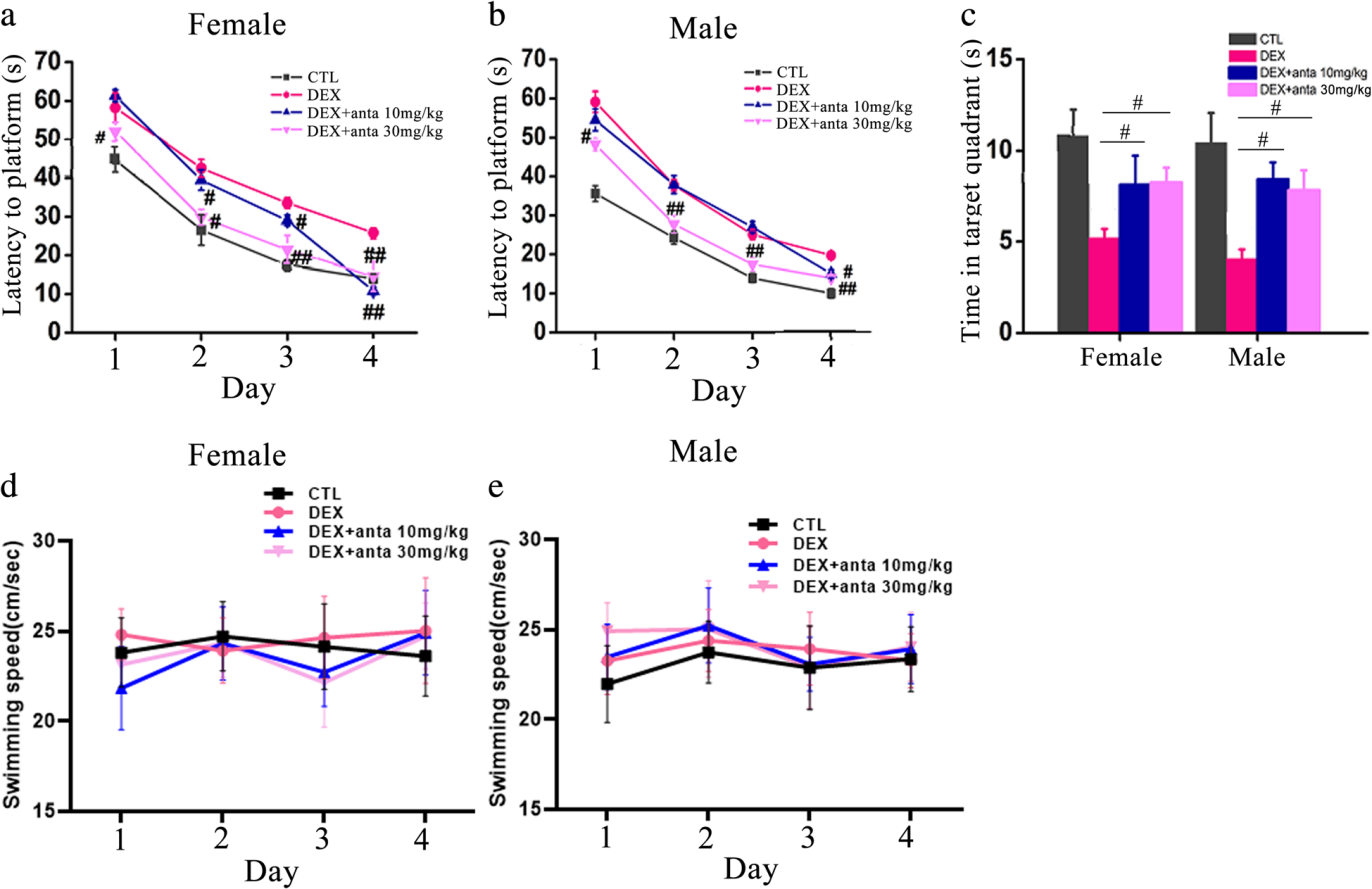
Antalarmin treatment improves spatial learning and memory in DEX offspring. DEX offspring of 2 days old were subcutaneously administered with antalarmin (10mg/kg/day, 30mg/kg/day) for 7 days as described in the “Materials and methods” section. Morris water maze test was performed on these rats in adulthood. **a**, **b** The escape latencies of female (**a**) and male (**b**) DEX offspring in the 4-day platform acquisition training. **c**, the time that spent in the correct platform quadrant in the probe trial after training. **d**&**e**, the swimming speed of the rats. Data are presented as mean ± SEM (*n*=12 in each group). #*P*<0.05, ##*P*<0.01 vs DEX. CTL: control; anta: antalarmin

Our previous study has shown CRH and CRHR1 expression is upregulated in hippocampus of rats with prenatal DEX exposure [20]. We then investigated whether CRHR1 signaling plays critical roles in impairment of learning and memory in the offspring with prenatal DEX exposure. The specific CRHR1 antagonist CP154526 and antalarmin were used to block the CRH signaling. Prenatal DEX offspring at two-days old received CRHR1 antagonist CP154526 or antalarmin treatment for a week. DEX offspring with CP154526 or antalarmin treatment in adulthood showed an improvement in spatial learning and memory compared with those with vehicle treatment. In the acquisition trial, both female and male DEX offspring with CP154526 (10 and 30mg/kg/day) treatment displayed shorter escape latencies than those with vehicle treatment on days 1–4 (Fig. 3a&b). In the probe trial, DEX offspring with CP154526 (10 and 30mg/kg/day) treatment spent longer amount time in staying in correct quadrant than those with vehicle treatment (Fig. 3c). No significant difference in swimming speed among CP154526 and vehicle treatment groups was displayed (Fig. 3d&e). Antalarmin treatment (30mg/kg/day) improved the acquisition of spatial learning compared with vehicle treatment in the tests on days 1–4 (Fig. 4a&b) and increased the time in staying in correct quadrant compared with vehicle treatment in both of female and male DEX offspring (Fig. 4c). However, antalarmin at the dosage of 10mg/kg/day did not significantly improve the acquisition of spatial learning test on days 1–3. There was no difference in swimming speeds among all the groups (Fig. 4d&e).

### CRHR1 antagonist treatment can ameliorate prenatal DEX exposure-induced decreased pre-and post-synaptic signals in the hippocampus

**Fig. 5:**
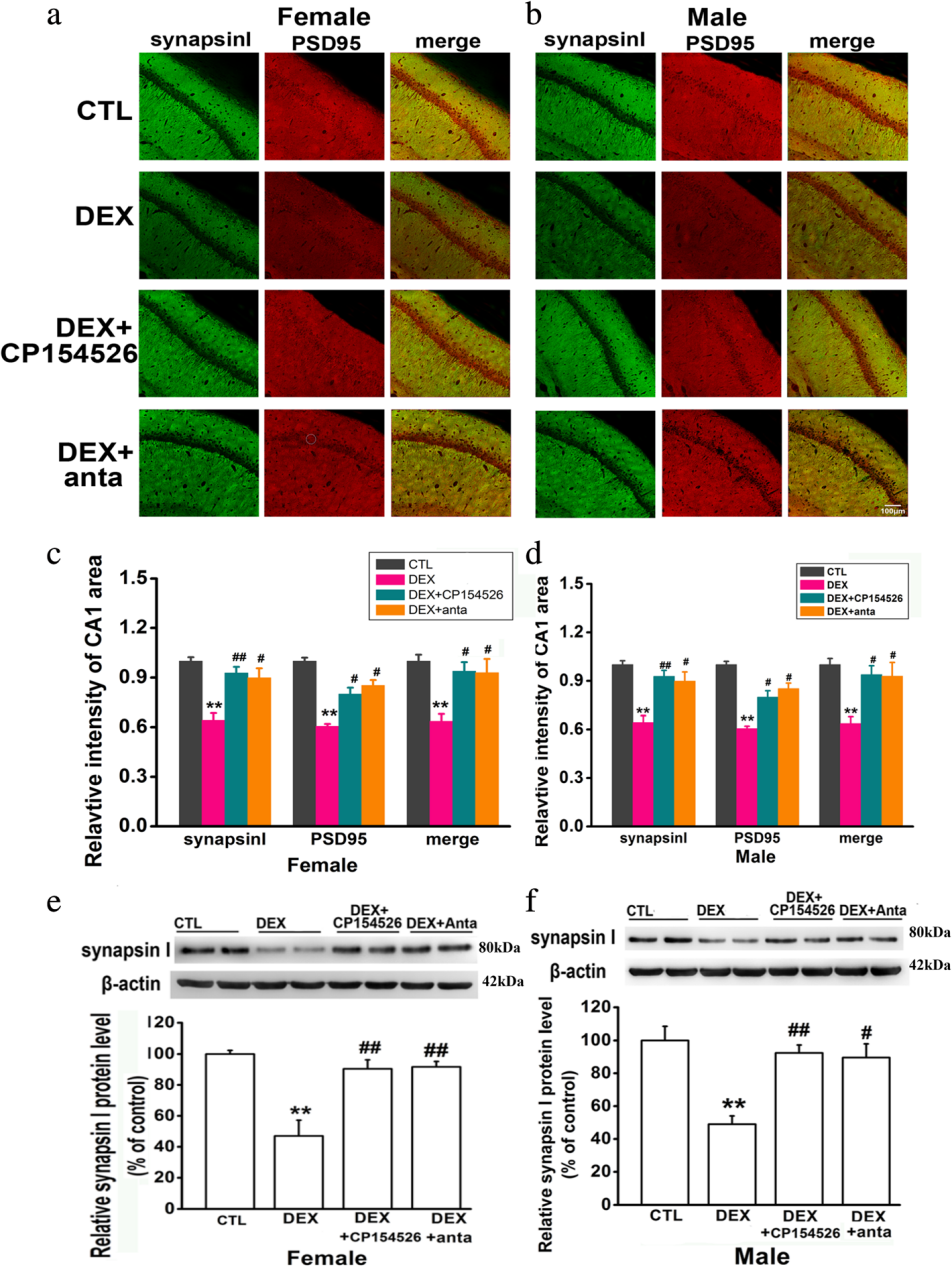
CRHR1 antagonists increase synapsin I and PSD95 signals and synapsin I level in the hippocampus of the rats with prenatal DEX exposure. DEX offspring of 2 days old were subcutaneously administered with CP154526 (30mg/kg/day) or antalarmin (30mg/kg/day) for a week. The hippocampi were collected after behavioral test under anesthesia. Synapsin I (green) and PSD95 (red) were used to label presynaptic and postsynaptic terminals in hippocampus, respectively. **a**, **b** Representative fluorescence images show synapsinI-and PSD95-labeled signals in CA1 area of the hippocampus of adult female (**a**) and male (**b**) DEX offspring. **c**, **d** Histogram summarized the effects of CRHR1 antagonists on the intensity of synapsin I and PSD95-labeled signals in CA1 area of female (**c**) and male (**d**) DEX offspring. Data are presented as mean ± SEM (*n*=4 in each group). **e**&**f**, protein level of synapsin I in female (**e**) and male (**f**) DEX offspring was determined by western blot. Representative protein bands are presented at top of histogram. Data are presented as mean ± SEM (*n*=8 in each group). **P<0.01 vs control; #*P*<0.05, ##*P*<0.01 vs DEX. CTL: control, anta: antalarmin

Synapsin I, a member of the family of phosphoproteins that cross-links synaptic vesicles to cytoskeletal elements within the presynaptic terminals, and PSD95, a predominant component of postsynaptic densities (PSDs), were used as the markers of presynaptic and postsynaptic signals, respectively. Since CA1 region has been widely reported to play a key role in spatial learning and memory [34, 35], we compared synapsin I- and PSD95-labled signals in CA1 region of the hippocampus between control and prenatal DEX groups. As shown in Fig. 5a–d, prenatal DEX exposure decreased synapsin I intensity by 54.1±4.1% in females and 65.7±3.9% in males compared with control treatment, respectively. PSD95 positive signals were decreased by 63.7±3.4% in prenatal DEX females and 59.8±3.5% in prenatal DEX males, respectively. Accordingly, the intensity of synapsin I and PSD95 colocalized synapses was decreased by 58.7±4.6% in prenatal DEX females and 64.9±4.2% in prenatal DEX males compared with corresponding control offspring. CP154526 (30mg/kg/day) and antalarmin (30mg/kg/day) treatment increased synapsin I- and PSD-labeled signals in both of females and males compared with vehicle treatment. The intensity of synapsin I and PSD95 colocalized synapses was also increased in DEX offspring treated with CP154526 (30mg/kg/day) and antalarmin (30mg/kg/day) treatment compared with those with vehicle treatment.

Western blotting analysis showed that prenatal DEX exposure caused a decrease in hippocampal synapsin I expression level compared with prenatal control treatment in both females and males (Fig. 5e&f). DEX offspring with CRHR1 antagonist treatment showed increased levels of synapsin I in the hippocampus compared those with those with vehicle treatment. CP154526 (30mg/kg/day) and antalarmin (30mg/kg/day) significantly increased hippocampal synapsin I expression level compared with vehicle treatment in both of female and male DEX offspring.

### Decreased CXCL5 expression in the hippocampus contributes to spatial learning and memory deficit caused by prenatal DEX exposure

As mentioned, we previously shown that CXCL5 is essential for synapses formation in hippocampal neurons in vitro, and CRHR1 suppression of synaptic formation in cultured hippocampal neurons is associated with decreased CXCL5 production [29]. We therefore explored whether CXCL5 signaling was involved in impaired spatial learning and memory caused by prenatal DEX exposure.

**Fig. 6:**
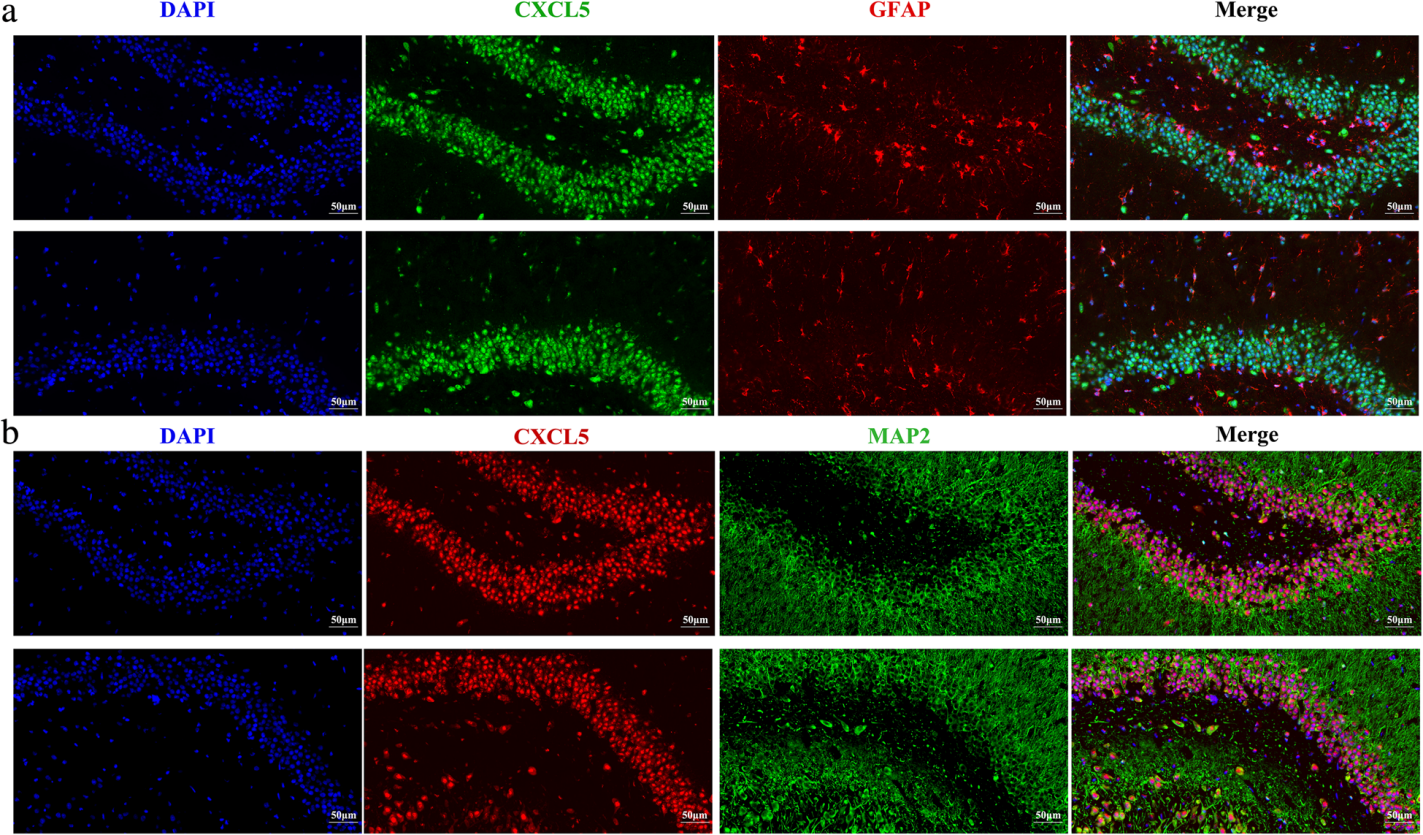
CXCL5 localization in the hippocampus of rats. The brain tissues were then fixed in 4% paraformaldehyde in phosphate-buffered saline (PBS) for 24 h at 4 °C. Immunofluorescence analysis was applied to show CXCL5 localization in rat hippocampus. **a**, representative fluorescence images show the co-localization of CXCL5-positive staining and GFAP-positive staining. **b**, representative fluorescence images show the co-localization of CXCL5-positive staining and MAP2-positive staining. Arrows indicate the cells with double staining

We investigated CXCL5 expression in the hippocampus of rats. It was found that CXCL5-positive staining was identified in CA region of hippocampus (Fig. 6). Of note, CXCL5-positive staining was mainly co-localized with MAP-2-positive staining cells (Fig. 6b). Some CXCL5-positive staining cells were also GFAP-positive stained (Fig. 6a), suggesting that CXCL5 is expressed in both of neurons and glial cells in the hippocampus. In addition, CXCL5-positive staining was found in cytoplasm and nucleus of neurons and glial cells.

**Fig. 7:**
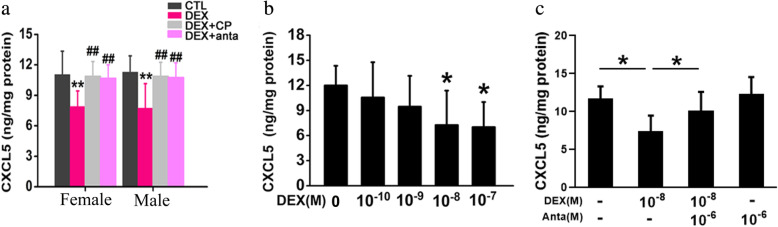
The effects of DEX treatment on CXCL5 level in the hippocampus in vivo and in vitro. **a**, DEX offspring of 2 days old were subcutaneously administered with CP154526 (30mg/kg/day) or antalarmin (20mg/kg/day) for a week. The protein level of CXCL5 in the hippocampus was determined by ELISA. Data are presented as mean ± SEM (*n*=8 in each group). ** *P<0.01* vs control; # *P<0.05*, ## *P<0.01* vs DEX. **b**&**c**, cultured hippocampal slices were treated with various concentration of DEX in the absence (**b**) and presence of CRHR1 antagonist (**c**) for 24h. Data are presented as mean ± SEM (*n*=4 cultures).*P<0.05 vs vehicle or indicated. CTL: control, anta: antalarmin, CP:CP154526

ELISA showed that prenatal DEX exposure resulted in a decrease in hippocampal CXCL5 content in both sex. Treatment of DEX offspring with CP154526 (30mg/kg/day) increased CXCL5 content in adulthood compared with vehicle treatment. Antalarmin (30mg/kg/day) treatment also increased hippocampal CXCL5 content compared with vehicle (Fig. 7a). In the model of cultured hippocampal slices, we found that DEX treatment suppressed CXCL5 production in a dose-dependent manner (Fig. 7b). CRHR1 antagonist antalarmin prevented DEX inhibition of CXCL5 production in cultured hippocampal slices (Fig. 7c).

**Fig. 8:**
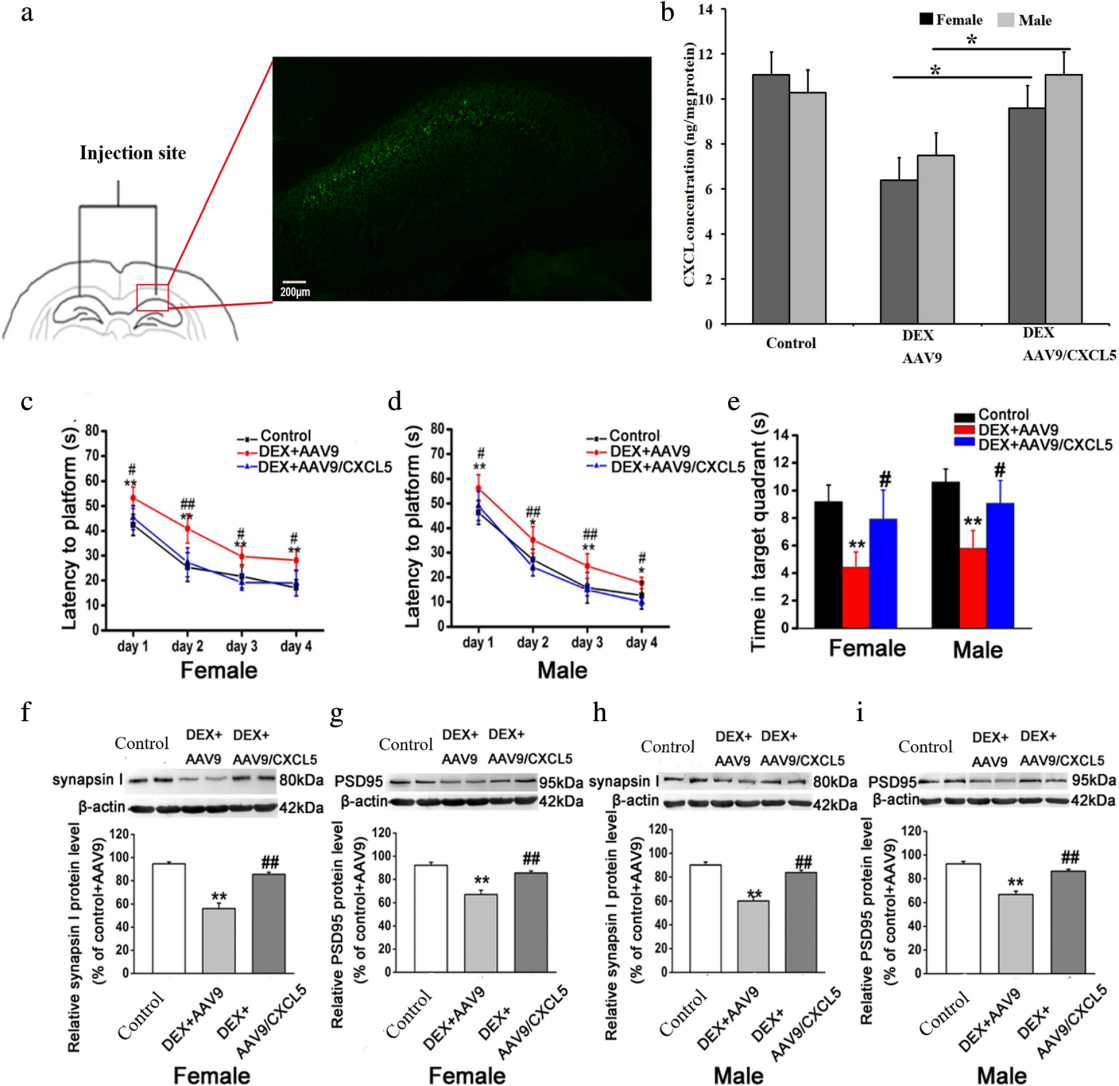
CXCL5 contributes to hippocampal CRH/CRHR1 mediated learning and memory deficits caused by prenatal DEX exposure. AAV9/CXCL5 or AAV9 was bilaterally injected into CA1 region of hippocampus of prenatal DEX. Four weeks after injection, the rats were tested for Morris water maze, and then sacrificed and hippocampus was dissected for determination of synaptic protein expression and CXCL5 concentration. **a** shows the injection site and immunofluorescence image of AAV9 infection in rat hippocampus. **b** shows CXCL5 level in DEX offspring with intra-hippocampal injection of AAV9 and AVV9/CXCL5. Data are presented as mean±SEM (*n*=6). *P<0.05. **c-e** Morris water maze tests in DEX offspring with AAV9 infusion. **c**&**d** show the escape latencies of female (**c**) and male (**d**) DEX offspring in the 4-day platform acquisition training. **e** shows the time that DEX offspring spent in the correct quadrant in the probe trial stage. **f**-**i**, the protein levels of synapsin I and PSD95 in hippocampus. Hippocampal tissue was obtained from adult female (**f**&**g**) and male (**h**&**i**) offspring injected with AAV9 or AAV9/CXCL5 as indicated. Representative protein bands are presented at top of histogram. Data are presented as mean ± SEM (n=6 in each group). *P<0.05 vs DEX+AAV9; **P<0.01vs control; # *P*<0.05, ## *P*<0.01 vs DEX+AAV9

We then elucidated whether intra-hippocampal injection of AAV9 encoding CXCL5 could improve spatial learning and memory in DEX offspring. Adult control and DEX offspring received intra-hippocampal injection of AAV9 or AAV9 carrying CXCL5 gene (Fig. 8a). Four weeks later, green fluorescence in CA1 region of the hippocampus was found in these rats, indicating that AAV9 infected this region. Hippocampal CXCL5 content was increased in prenatal DEX rats with intra-hippocampal injection of AAV9/CXCL5 compared with those with injection of AAV9 (Fig. 8b). Both female and male DEX offspring with injection of AAV9/CXCL5 showed improvement of spatial learning and memory in MWM test as evidenced by decreased escape latency in finding the hidden platform and increased time in the correct quadrant compared with those with injection of AAV9 vector (Fig. 8c–e). We then examined synapsin I and PSD95 expression level in the hippocampus. It was shown that intra-hippocampal injection of AAV9/CXCL5 increased synapsin I and PSD95 expression level compared with injection of AAV9 vector (Fig. 8f–i).

## Discussion

The present study demonstrated that prenatal DEX exposure impaired spatial learning and memory and resulted in a decrease in synaptic signals and synaptic protein expression in the hippocampus, suggesting that impaired learning and memory is associated with decreased synaptic network in the hippocampus. Treatment of newborn DEX offspring with CRHR1 antagonists improved spatial learning and memory and increased synaptic signals and synaptic protein level in the hippocampus of these rats in adulthood. Prenatal DEX exposure led to a decrease in CXCL5 level in the hippocampus. Intra-hippocampal injection of AAV9/CXCL5 rescued hippocampal-dependent spatial learning and memory deficiency and increased synaptic protein expression in the rats exposed to DEX prenatally.

Increasing bodies of evidence have indicated that early life experiences shape developing neuronal pathways and long-term plasticity. As mentioned, a number of studies have shown that prenatal exposure to GCs, either by sGC administration or as a result of maternal stress, impairs learning and memory and long-term potentiation (LTP) in rodents [15–17, 30, 36, 37]. In consistent with these studies, we also found that prenatal DEX exposure impaired spatial learning and memory. Some studies reported that the rodent model with early life stressful experience exhibits reduced dendritic complexity in hippocampal neurons in the dentate gyrus (DG), CA1, and CA3 area [25–27, 30]. Here, we showed that prenatal DEX exposure suppressed synaptic related protein synapsin I and PSD 95 signals in the hippocampus. Interestingly, CRHR1 antagonist treatment of DEX offspring not only improved spatial learning and memory but also increased synapsin I and PSD 95 signals in the hippocampus. Thus, it might let us indicate that decreased synaptic formation in the hippocampus contributes to spatial learning and memory deficit induced by prenatal DEX exposure.

The mechanisms responsible for prenatal DEX exposure-induced impairment of learning and memory remain largely unknown. As mentioned, CRH is the key mediator in stress-related impairment of learning and memory [25–27]. The studies by Fenoglio et al. [38] and Ivy et al. [25] have shown that CRH expression in the hippocampus is persistently increased in mice exposed to stress during early postnatal development. Of note, our previous studies have shown that prenatal DEX exposure enhances CRH and CRHR1 expression in the hippocampus, and increased expression of CRH and CRHR1 in the hippocampus occurs from embryonic phase to adult [20]. We therefore intended to block CRH/CRHR1 signaling by treatment of DEX offspring with CRHR1 antagonist early in life. The results showed that the treatment of newborn DEX offspring with CRHR1 antagonists improved spatial learning and memory of these rats in adulthood. Interestingly, we previously have demonstrated that CRHR1 antagonist treatment of new born DEX offspring decreases hippocampal CRH/CRHR1 system of these rats in adulthood [20], suggesting that CRHR1 antagonist reverses persistently increased CRH/CRHR1 system in hippocampus. Taken together, it indicates that increased hippocampal CRH/CRHR1 system play the critical role in prenatal DEX exposure-induced spatial learning and memory deficits.

Prior studies have shown that CRH/CRHR1 is involved in synaptic formation in hippocampus. For instance, CRHR1 knockout and blockage significantly attenuates the detrimental effects of chronic stress on dendritic arborization and rapid loss of apical dendritic spines in hippocampus [27, 30]. We previously showed that CRH suppresses synaptic formation as evidenced by decreased synapsin I and PSD95 signals in cultured hippocampal slices and neurons via CRHR1 [30]. Here, we found that CRHR1 antagonist treatment increased hippocampal synapsin I and PSD95 signals in DEX offspring might contribute to impaired synaptic network in the hippocampus of DEX offspring. As mentioned, CRHR1 antagonist treatment can reverse persistently increased CRH/CRHR1 system in the hippocampus of these rats. It would let us suggest that impaired synaptic network caused by prenatal DEX exposure might be attributed to persistently increased CRH/CRHR1 signaling in the hippocampus.

The present study found that prenatal DEX exposure decreased CXCL5 level in the hippocampus and infusion of AAV9 carrying CXCL5 gene into CA1 region improved spatial learning and memory in DEX offspring, which suggest that reduced CXCL5 signaling contributes to spatial learning and memory deficits caused by prenatal sGC exposure. Merabova et al. [39] reported that CXCL5 can act as one of glia derived chemokines to play a critical role in neuronal survival. We have previously demonstrated that CXCL5 is a key factor for synaptic formation in cultured hippocampal neurons [29]. In the present study, we found that prenatal DEX exposure decreased CXCL5 level in the hippocampus and administration of AAV9 carrying CXCL5 gene into CA1 region increased synaptic protein expression in DEX offspring. Thus, it might indicate that decreased CXCL5 in the hippocampus contribute to impaired synaptic network caused by prenatal DEX exposure. Of note, a number of cytokines and chemokines have been shown to be involved in regulation of synaptic transmission [40–43]. Thus, CXCL5 regulation of spatial learning and memory might be associated with functional changes in synapses. In addition, we found that CXCL5-positive staining was mainly found in neurons in the hippocampus, which indicates that CXCL5 might be involved in various functions of these neurons. Although glial cells are the main sources of cytokines in nervous system, many studies have demonstrated that neurons are able to produce cytokines [44–47]. Our study also provides the evidence that neurons are one of source of cytokines in brain.

Our previous study has shown that DEX offspring display increased circulatory GC level from early life to adult, and treatment of newborn DEX offspring with CRHR1 antagonist can reverse persistently increased GC level in circulation of these rats [20]. Thus, the improving effect of CRHR1 antagonist treatment on spatial learning and memory might also be attributed to disruption of persistently increased circulatory GC level in DEX offspring. Similarly, persistently increased circulatory GC level also contributes to decreased CXCL5 production in the hippocampus of DEX offspring.

We found that blockage of CRHR1 signaling increased hippocampal CXCL5 level in DEX offspring, and CRHR1 antagonist prevented DEX-induced reduction of CXCL5 production in cultured hippocampal slices. These data might let us suggest that CRH/CRHR1 signaling is involved in reduced CXCL5 production in the hippocampus caused by prenatal DEX exposure. Although our previous study showed that CRH suppresses CXCL5 secretion in cultured glia cells [29], CRH/CRHR1 modulates CXCL5 production in neuron or glia in the hippocampus remains to be defined in intact animal model.

There are a number of limitations in the present study. For instance, [1] specific manipulations should be applied to show synapse morphology in hippocampus of the rats with prenatal DEX exposure and those with CXCL5 intervention, [2] the cellular source of CXCL5 that is involved in regulation of learning and memory remains to be elucidated, and [3] CXCL5 modulation of synapse formation remains to be confirmed in intact animal model by using molecular approaches such as conditional CXCL5 knockout mice.

## Conclusion

In summary, prenatal sGC exposure impairs spatial learning and memory in both male and female offspring and concurrently reduces CXCL5 production and synaptic protein expression in the hippocampus. Increased CRH/CRHR1 system and decreased CXCL5 expression in the hippocampus contributes to spatial learning and memory deficits caused by prenatal sGC exposure. Enhanced CRH/CRHR1 signaling contributes to reduced CXCL5 production in the hippocampus due to prenatal sGC exposure. Our data provide the potential molecular basis of prenatal GC programming spatial learning and memory.

## Data Availability

All data generated or analyzed during this study are available from the corresponding author upon rational request.

## Abbreviations

CRH: Corticotropin-releasing hormone
CRHR1: CRH receptor type 1
CXCL5: C-X-C chemokine ligand 5
DEX: Dexamethasone
DG: Dentate gyrus
ELSIA: Enzyme-linked immunosorbent assay
GC: Glucocorticoid
GD: Gestational day
LTP: Long-term potentiation
MWM: Morris water maze
SDS: Sodium dodecyl sulfate
sGC: Synthetic GC
SPSS: Statistical Package for Social Sciences
TBST: Tris-buffered saline/Tween 20
